# Antihyperuricemic and xanthine oxidase inhibitory activities of *Tribulus arabicus* and its isolated compound, ursolic acid: *In vitro* and *in vivo* investigation and docking simulations

**DOI:** 10.1371/journal.pone.0202572

**Published:** 2018-08-16

**Authors:** Eman Abu-Gharbieh, Naglaa G. Shehab, Ihab M. Almasri, Yasser Bustanji

**Affiliations:** 1 Department of Clinical Sciences, College of Medicine, University of Sharjah, Sharjah, United Arab Emirates; 2 Department of Pharmacology and Toxicology, Dubai Pharmacy College, Dubai, United Arab Emirates; 3 Department of Pharmaceutical Chemistry and Natural Products, Dubai Pharmacy College, Dubai, United Arab Emirates; 4 Department of Pharmacognosy, Faculty of Pharmacy, Cairo University, Cairo, Egypt; 5 Department of Pharmaceutical Chemistry and Pharmacognosy, Faculty of Pharmacy, Al Azhar University, Gaza, Palestine; 6 Department of Biopharmaceutics and Clinical Pharmacy, Faculty of Pharmacy, The University of Jordan, Amman, Jordan; 7 Hamdi Mango Center for Scientific Research, The University of Jordan, Amman, Jordan; University of South Alabama Mitchell Cancer Institute, UNITED STATES

## Abstract

**Background:**

Hyperurecemia is usually associated with gout and various metabolic arthritis disorders. Limited medications are available to manage such conditions. This study aimed to isolate the triterpenes constituent of the plant and to assess xanthine oxidase (XO) inhibitory and antihyperuricemic activities of *Tribulus arabicus* ethanolic extract, its fractions and the isolated compound using *in vitro and in vivo* approaches.

**Methods:**

The ethanolic extract, fractions; n-hexane, chloroform and n-butanol and the isolated compound (ursolic acid) were evaluated *in vitro* for their XO inhibitory activity. Those that demonstrated significant activity were further evaluated for their antihyperuricemic activity on potassium oxonate-induced hyperuricemia in mice.

**Results:**

The ethanolic extract was found to be safe up to 5000 mg/kg. The extract and its n-hexane fraction exhibited significant inhibitory activity on XO, whilst only a modest reduction in the enzymatic activity was noticed with n-butanol and chloroform fractions. Furthermore, administration of the ethanolic extract at low and high doses significantly reduced serum urate levels in mice by 31.1 and 64.6% respectively. The isolated active constituent, ursolic acid, showed potent XO inhibition activity (Half maximal inhibitory concentration, IC_50_ = 10.3 μg/mL), and significantly reduced uric acid level *in vivo* by 79.9%. Virtually, the binding mode of ursolic acid with XO was determined using molecular docking simulations.

**Conclusions:**

The activity of the ethanolic extract of *T*. *arabicus* and its n-*hexane* fraction can be attributed to the isolated compound, ursolic acid. Ursolic acid has good hypouricemic activity and therefore has high potential to be used for the treatment of gout and hyperuricemia-related diseases.

## Introduction

Gout or metabolic arthritis is an inflammatory disease, which usually targets the joints and is caused by an abnormal build-up of uric acid in the blood. Xanthine oxidase (XO) is known to convert purine from protein-rich foods, such as organ meats and fish, to its metabolic by-product, uric acid. XO is linked to multiple human diseases such as ischemia-reperfusion injury and the development of various cardiovascular and inflammatory diseases, due to its ability to generate reactive oxygen species [1]. Treatment of gout involves the use of therapeutic agents, such as xanthine oxidase inhibitors (XOI) that act by blocking the conversion of purine into uric acid [2,3].

Allopurinol is a well-known xanthine oxidase inhibitor, and is widely used in the therapeutic and clinical management of gout [4,5]. However, allopurinol has many intolerable side effects that can sometimes be fatal. Examples of such severe adverse drug reactions of allopurinol include: hypersensitivity syndrome [2,6], liver function abnormalities [7,8] and Toxic Epidermal Necrolysis syndrome (TENS) [5].

The use of herbal medicinal has increased extremely now and many natural products are familiarized into the market and public health problems. Over 80% of the people worldwide relying on natural products for treatment of some diseases. On the other hand, many of natural products still untested and with an inadequate information of their mode of action and their safety [9]. Triterpenes are among the most abundant natural products especially pentacyclic triterpenes that have received much attention and are being marketed as therapeutic agents or dietary supplements around the world [10].

The United Arab Emirates houses many uninvestigated species of desert plants, including shrubs and herbs, some of which could be explored for their potential therapeutic uses. Genus *Tribulus* belongs to family Zygophyllaceae, is native to warm temperate and tropical regions. The Latin name *Tribulus* originally meant the caltrop (a spiky weapon) [11].

The plants belonging to genus *Tribulus* traditionally are known in preventative medicine as an enhancer of testosterone and are taken as a supplement to enhance sexual urges among both men and women [12,13]. Genus *Tribulus* can be of great use to people who need help against infections, as reported in a recent Iraqi study [14]. Moreover, the genus has shown 'significant' protection against the deposition of kidney stone-forming material, in addition to inhibiting elevations in blood urea levels. Phytochemical investigations on genus *Tribulus* revealed the presence of a variety of chemical constituents that are medicinally important such as flavonoids, flavonol glycosides, beta-sitosterols or stigma, steroidal saponins, triterpenes and alkaloids [15].

The aqueous extract of *Tribulus terrestris* significantly reduced the excretion of oxalate, calcium, and phosphate in addition it decreased the levels of blood urea nitrogen, uric acid and creatinine in rats’ serum [12,16]. According to these findings, we decided to evaluate *Tribulus arabicus*, locally known as Zahar, Abu Drais, Sharshar and Hasak, for its ability to inhibit XO activity and thus reduce serum uric acid level. *Tribulus arabicus* is a perennial herb with grey-green leaves and with large yellow flowers (1.5–2.0 cm) [17], and it is reported to have significant antioxidant potential [18]. There is little information in the literature either on the biological activity of this species or its chemical constituents.

This study aimed to isolate the triterpenoidal compounds from *T*. *arabicus*, assess xanthine oxidase (XO) inhibitory and antihyperuricemic activities of the plant ethanolic crude extract, fractions and the isolated compound using both *in vitro* and *in vivo* models.

## Materials and methods

### Chemicals

N-Hexane, chloroform, n-butanol and ethanol were purchased from Fisher Scientifics (UK). Silica Gel 60, Xanthine oxidase, xanthine, allopurinol, and uric acid assay kit were purchased from Sigma (USA). All other chemicals were of analytical grade. Melting point was determined on Electrothermal 9100 equipment. Mass spectrum was measured on a Jeol Mass Spectrometer SSQ 7000, Digital DEC 300. NMR spectrum was measured in DMSO. ^1^H–NMR spectrum was obtained at 400 MHz on a JEOL GX-400 spectrometer with the chemical shifts (δ ppm) expressed relative to TMS as internal standard. Precoated silica gel 60 F254 (Merck, Darmstadt, Germany) was used for the TLC analysis. Vacuum liquid chromatography (VLC) was performed on silica gel 60 GF (Merck, Darmstadt, Germany)

### Plant material

Arial parts of *T*. *arabicus* plant were collected during October 2015 from Muhaisnah desert, Dubai, UAE. The plant was kindly identified by Zayed Complex for Herbal Research and Traditional Medicine in Abu-Dhabi. Voucher specimens were kept at the Herbarium of Dubai Pharmacy College (#5-10-15). The plant was air-dried in shade and was powdered.

### *In vitro* evaluation

#### Plant extraction

The air-dried powdered plant (2.0 kg) was exhaustively extracted by cold maceration in ethanol (8 L x 2). The solvent was evaporated under reduced pressure at 50°C yield 90 g residue. Portion from the crude residue (70 g) was successively fractionated by n-hexane, chloroform and n- butanol. The solvent, in each case was evaporated to yield 10.0, 18.6 and 3 g respectively. The extractives subjected to specific chemical tests and TLCs screening for qualitative identification of their components.

#### Isolation of ursolic acid from hexane fraction

Six grams from the residue of hexane extract were fractionated on vacuum liquid chromatography, VLC (30 ×3.5 cm) using silica Gel 60. Gradient Elution was carried out by using mixtures from hexane/chloroform and chloroform/ethyl acetate. Fractions (100 ml each) were collected and tested for their constituents by thin-layer chromatography (TLC) using system 1 (benzene-ethyl acetate, 86:14) and system 2 (chloroform—methanol, 9.5:0.5). The spots were visualized under UV with or without ammonia vapor and by spraying the TLCs plates with *p-*aniseldehyde. Ten fractions were pooled together and Fraction 3 (Chloroform–ethyl acetate, 90:10, 1.0 g) was chosen, purified and crystalized by methanol-water to give ursolic acid.

#### Xanthine oxidase inhibitory activity assay

Xanthine oxidase inhibitory activity was assayed spectrophotometrically at 290 nm using Ultraviolet–visible (UV–VIS) microplate reader as previously described method [19]. The tested samples were dissolved initially in dimethyl sulfoxide (DMSO) and subsequently were diluted with phosphate buffer (pH = 7.5) to a final concentration containing less than 1% DMSO (v/v). Seven different concentration ranges (5, 10, 20, 50, 100, 200 and 300 μg/mL) of each tested sample were used to determine the concentration that inhibits 50% of the XO enzyme activity (IC_50_). A mixture consisting of 50 μL of test solution, 35 μL phosphate buffer and 30 μL of XO solution freshly prepared (0.1 U/mL) was pre-incubated for 15 min at 25°C. The reaction was then initiated by adding 60 μL of xanthine solution (150 μM) into the mixture. The final mixture was incubated for 30 min at 25°C. The reaction was stopped by adding 25 μL of HCl solution (1N) prior to measuring the absorbance. Allopurinol was used as a positive control. The negative control (blank) was prepared similarly, but by adding HCl solution before the substrate.

All analyses were run in triplicate, and the inhibition percentages were calculated using the below equation, in which α is the activity of XO without test extract and β is the activity of XO with test extract.

$$Percentageof\;XO\;inhibition=(1-\frac{\beta }{\alpha })*100$$ (1)

### Biological evaluation

#### Animals

BALB/c male mice weighing 25–32 g, approximately 11 weeks old, were used for acute toxicity and antihypeuricemic studies. All animals were maintained under standard conditions, fed with regular diet and water supplied *ad libitum*. Animals were accommodated for 7 days preceding the experiments. Experimental protocols were approved by the Ethical Research Committee of Dubai Pharmacy College, Dubai United Arab Emirates and performed in accordance with the ethical standards of laboratory animals [20].

#### Acute oral toxicity

Lethal Dose, 50% (LD_50_) for the plant extract was determined according to Probit test [21]. LD_50_ of ursolic acid was previously reported to be 9260 mg/kg [22]. Mice were divided into five groups of 10 animals each, and they received different oral doses of the ethanolic crude extract ranges from 250 to 5000 mg/kg. Over three days, the animals were observed for any signs of morbidity or abnormal behavior and their death was recorded.

#### Mice model of hyperuricemia

Hyperuricemia was induced by injecting the animals with the uricase inhibitor, potassium oxonate (PO), intraperitoneally at dose of 250 mg/kg. All injections were given one hour before administrating the tested samples throughout the study period [23].

#### Animal experimental protocol

Based on the *in vitro* results, the active extract, fractions and/or isolated compound were selected for further *in vivo* evaluation. Mice were randomly divided into nine groups of eight animals each. All animals fasted for six hours before the treatments. The first group served as control and did not receive treatment nor potassium oxonate injection. The second group was the hyperuricemia group, and animals were injected with potassium oxonate only. Both groups were given 1% carboxymethyl cellulose (CMC) to eliminate any vehicle-related variations. The third group served as positive control and the animals received allopurinol orally at dose of 20 mg/kg. The fourth and fifth groups received the ethanolic crude extract at two different doses of 100 and 200 mg/kg respectively. The six and seventh groups received n-hexane fraction at doses of 50 and 100 mg/kg. The last two groups received the isolated compound, ursolic acid, at dose of 5 and 10 mg/kg, respectively. All treatments were given orally once daily for 5 days.

Blood samples were collected from the tail vein of the mice one hour after the final administration. To separate the serum, the blood was allowed to clot for approximately one hour at room temperature then centrifuged at 3000 r/min for 10 min. Serum uric acid levels were measured using assay kits. At the end of the treatment period, the animals were anaesthetized with isoflurane and then sacrificed by cervical dislocation.

#### Docking simulations

*In silico* investigation of the binding mode of ursolic acid with XO was carried out using docking simulations. The three-dimensional (3D) coordinates of bovine XO enzyme (Protein Data Bank, PDB ID code: 1N5X, Rs = 2.80 Ǻ**)** was obtained from the Protein Data Bank [24]. Hydrogen atoms were added to proteins using Discovery Studio (DS) visualizer templates for protein residues [25]. The receptor file, containing information about the location and characteristics of binding pocket, was created using the PDB2RECEPTOR utility program for converting a protein-ligand complex into a receptor. The chemical structure of ursolic acid was sketched in MarvinSketch [26] and saved in molfile format. Subsequently, an ensemble of low energy conformers was generated using OMEGA 2.5.1.4. software [27]. OMEGA rapidly generates conformational ensembles of small molecules using a fragment-based library to build initial models of structures by bringing together these fragment templates followed by rule-based torsion search stage.

The generated conformers, saved in Standard Delay File (SDF) format, were then docked into the binding site of XO using FRED 3.2.0.2 OEDocking software in the presence of explicit water molecules [28]. FRED docks pre-generated multiconformer molecules within a box enclosing the active site of a single receptor using an exhaustive search that systematically searches rotations and translations of each conformer of the molecule within the active site. The top scoring poses are optimized and assigned a final score using Chemgauss4.

#### Statistical analysis

The results were expressed as mean ± SEM (standard error of the mean). Data was analyzed by GraphPad Software version 6.00 (San Diego, CA). One-Way ANOVA followed by Bonferroni’s multiple comparison tests versus the control was performed. p-value less than 0.05 was considered significant. IC_50_ values for XO inhibition assays were calculated from the dose–response curves. Power analysis has been used to specify the minimum number of animals that can be used and still give valid scientific results using 95% confidence interval levels and power of 80%.

## Results and discussion

The powdered plant was extracted by cold maceration in ethanol with subsequent successive fractionation with hexane, chloroform and n-butanol. Preliminary phytochemical screening and TLCs investigation of the ethanolic extract and its fractions revealed the presence of steroids and triterpenes in hexane fraction and presence of triterpenes, flavonoids and alkaloids in the chloroform and *n*-butanol fractions which encourage the authors to choose the hexane fraction in order to isolate the ursolic acid compound and to evaluate the possible therapeutic use of this compound.

**Fig 1:**
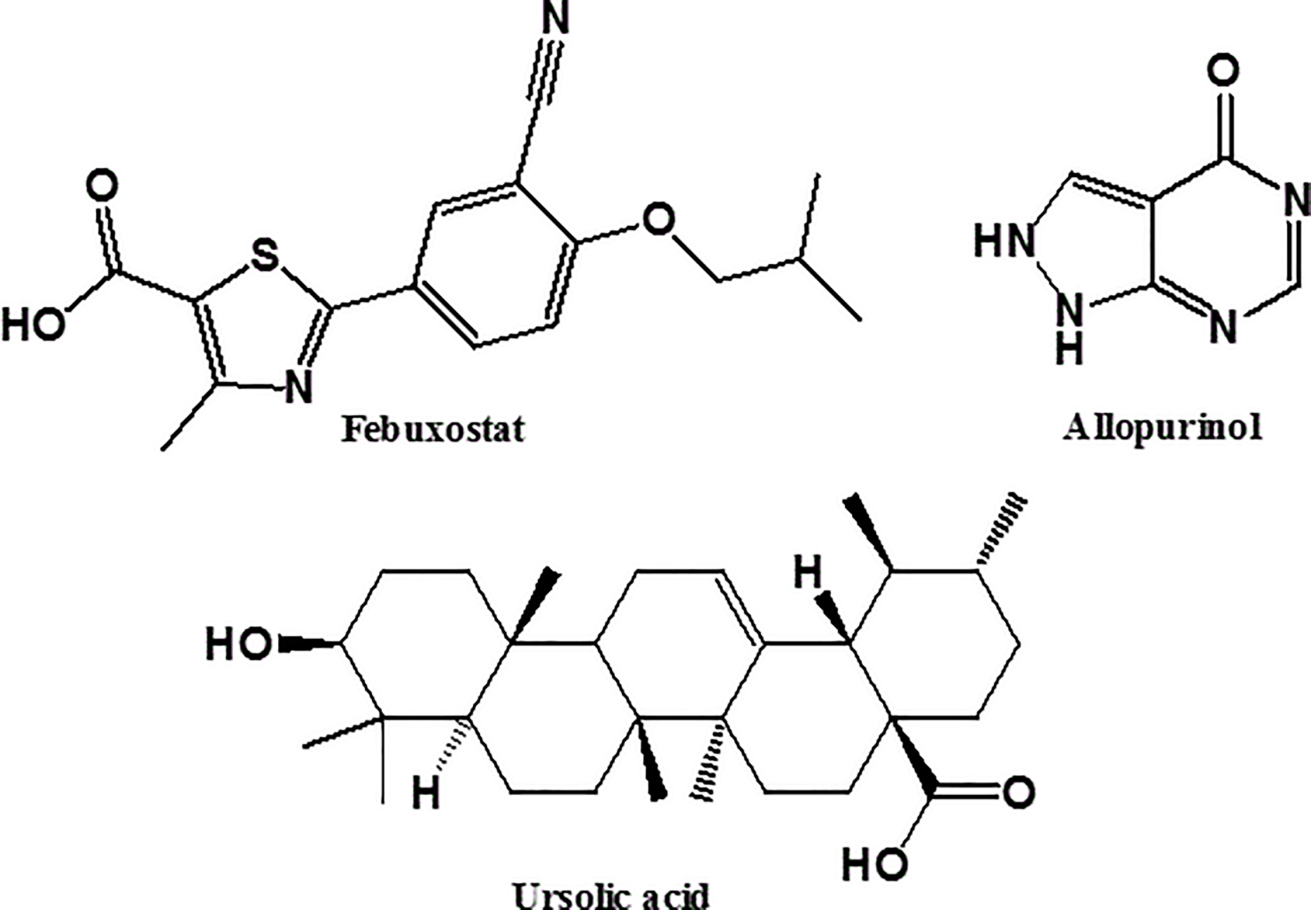
Chemical structures of febuxostat, ursolic acid and other xanthine oxidase inhibitors; allopurinol and febuxostat.

The hexane fraction was further chromatographed to yield 900 mg ursolic acid as white crystals which was identified based on the physical, chemical and spectral data. Ursolic acid (Fig 1) (C_30_H_48_O_3_) is a triterpene soluble in chloroform, crystallize in methanol-water; Retardation factor, R_f_ = 0.83 (system 2); gave positive test for sterols and/or triterpenes; melting point: 284°C, m/z 457, 439, 411 and 393; ^1^HNMR (400 MHz, DMSO): δH 0.69, 0.76, 0.82, 0.87and 1.04 (15 H, 5 s, all CH3), 0.83, (3H, d, J = 6.4 Hz, H-30), 0.92 (3H, d, J = 6 Hz. H-29), 1.30 (2H, m, H-21),1.46 (2H, m, H-16), 2.10 (d, 1H, J = 15 Hz, H-18), 3.32 (1H, dd, J = 10.8, 4.4 Hz, H-3), 5.13 (1 H, t; J = 3.6 Hz, H-12) as shown in S1 Fig [29].

### The *in vitro* XO-inhibitory activity of *T. arabicus*

The assay was conducted to investigate the ability of the extract, fractions and isolated compound to inhibit XO activity in comparison to allopurinol, a clinically used inhibitor. Results presented in Table 1 showed that the IC_50_ values of the ethanolic extract, n-hexane fraction and ursolic acid exhibited strong XO inhibitory activity *in vitro*, with 20.4, 12.6 and 10.3 μg/mL respectively. Butanol and chloroform fractions exhibited low inhibition activity against XO with IC_50_ values of 40.3 and 45.8 μg/mL respectively.

**Table 1:** Xanthine oxidase inhibitory activity of the tested samples (Mean ± SEM).

Tested sample	IC_50_ μg/mL
***T*. *arabicus* ethanolic extract**	20.4 ± 1.3
**n-Hexane fraction**	12.6 ± 2.1
**Chloroform fraction**	45.8 ± 2.4
**n-Butanol fraction**	40.3 ± 2.6
**Ursolic acid**	10.3 ± 3.5
**Allopurinol**	6.5 ± 2.6

### Acute oral toxicity of *T. arabicus* extract

An acute toxicity study showed that there was no lethality or any toxic behavior in the mice. The ethanolic crude extract of *T*. *arabicus* did not show any mortality or any visible symptoms of toxicity up to a dose of 5000 mg/kg body weight over three days of monitoring, indicating a high margin of safety.

### *In vivo* hypouricemic effect in hyperuricemic mice model

**Fig 2:**
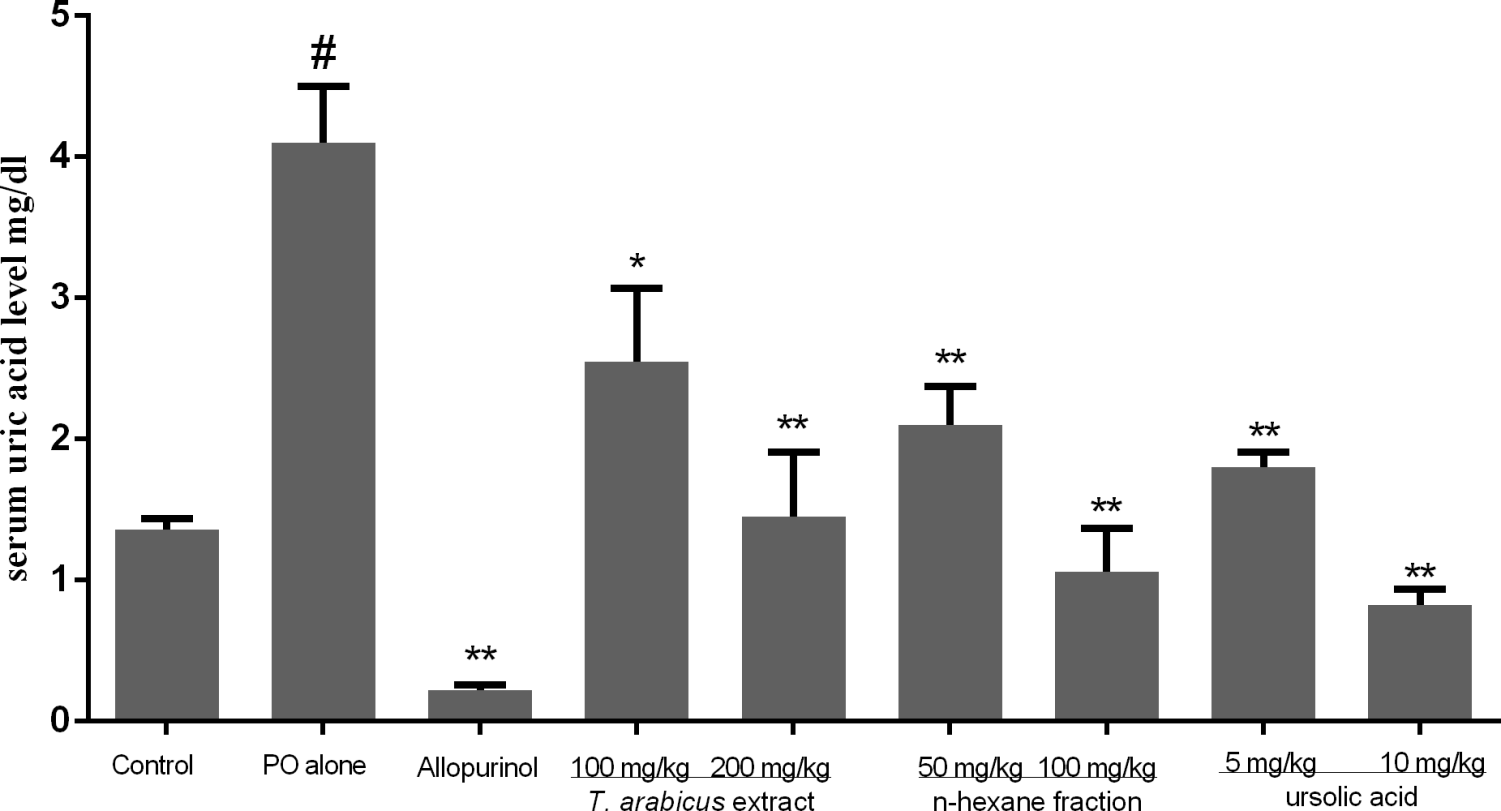
The uric acid-lowering effects of *T*. *arabicus* ethanolic crude extract and fractions; n-hexane and ursolic acid on mice with potassium oxonate-induced hyperuricemia in comparison with allopurinol. The results are presented as the mean ± SEM. #p < 0.001 versus the control group; *p < 0.01; **p < 0.001 versus the potassium oxonate treated group, n = 8.

In order to further confirm the capabilities of the plant extract, n-hexane fraction and ursolic acid to reduce the uric acid level *in vivo*, hyperuricemic animal model was developed using potassium oxonate (PO), a selective competitive uricase inhibitor, to induce hyperuricemia in mice [30]. Animals injected with PO developed hyperuricemia, as indicated by a significant increase in serum urate levels of 67% (p < 0.001) as shown in Fig 2. The doses used for further treatment were selected based on the LD_50_ value of the extract (<1/10^th^) and previously reported *in vivo* studies on ursolic acid [29,31,32]. Compared with the hyperurecemic mice, serum uric acid concentrations of groups treated with *T*. *arabicus* extract at doses of 100 and 200 mg/kg were reduced significantly in a dose-dependent manner by 31.1 and 64.6% respectively (Fig 2).

Interestingly, administration of n-hexane fraction in low and high doses reduced uric acid levels significantly, p < 0.001. In addition, treatment with the ursolic acid in low and high doses reduced uric acid level significantly (p < 0.001) by 56.1 and 79.9% respectively as shown in Fig 2. The *in vitro* and *in vivo* results suggested that inhibition of XO activity played an important role in the antihyperuricemic effects of *T*. *arabicus* extracts and ursolic acid.

Ursolic acid is a pentacyclic triterpenes widely presents in many plants. It has many biological activities such as antitumor, antioxidant, hepatoprotective, antimicrobial, anti-inflammatory, antiulcer, hypolipidemic and antiatherosclerotic [33]. Although low bioavailability of ursolic acid is a known drawback that hinders its effective oral application, a recent study has detected ursolic acis and its metabolites in mouse plasma and urine after single oral administration of ursolic acid saline solution [34]. Moreover, various *in vivo* studies reported various techniques to enhance ursolic acid oral bioavailability [35–37]. In our study, tested samples were prepared in surfactant, carboxymethyl cellulose, given over five days and mice were fasted over 6 hours before receiving the oral treatment to enhance oral absorption.

Interestingly, ursolic acid was also reported to have significant nephroprotective effect against gentamicin due to its ability to reduce serum urea, uric acid, creatinine and blood urea nitrogen [32]. Moreover, ursolic acid received Chinese’s patent for its promising application as antigout medication [38].

### Computational docking simulations

**Fig 3:**
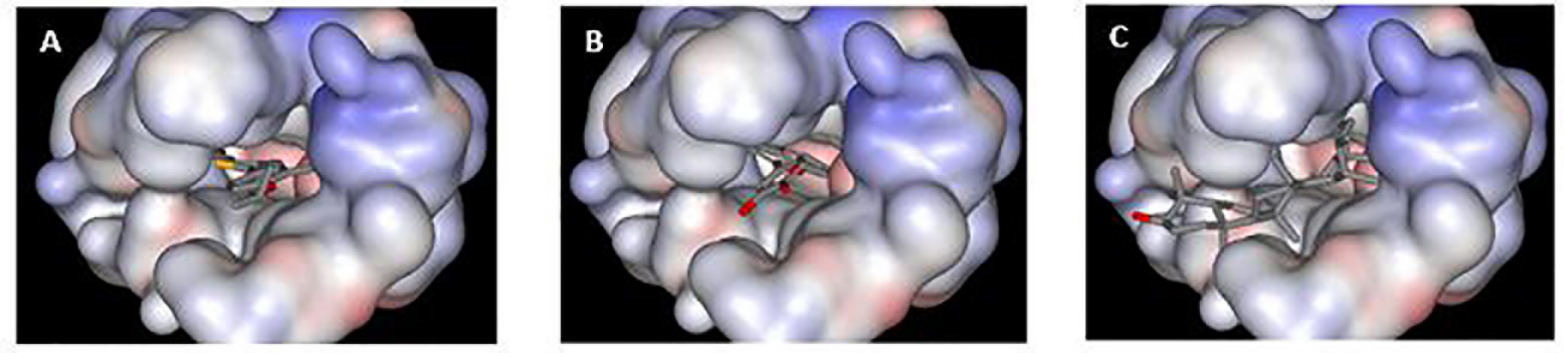
Comparison between binding modes of: Febuxostat (**A:** PDB code: **1N5X**) and Quercetin (**B**: PDB code: **3NVY)** (**B**) with ursolic acid(**C**) within the solvent-accessible surface area of the binding site of bovine xanthine oxidase.

**Fig 4:**
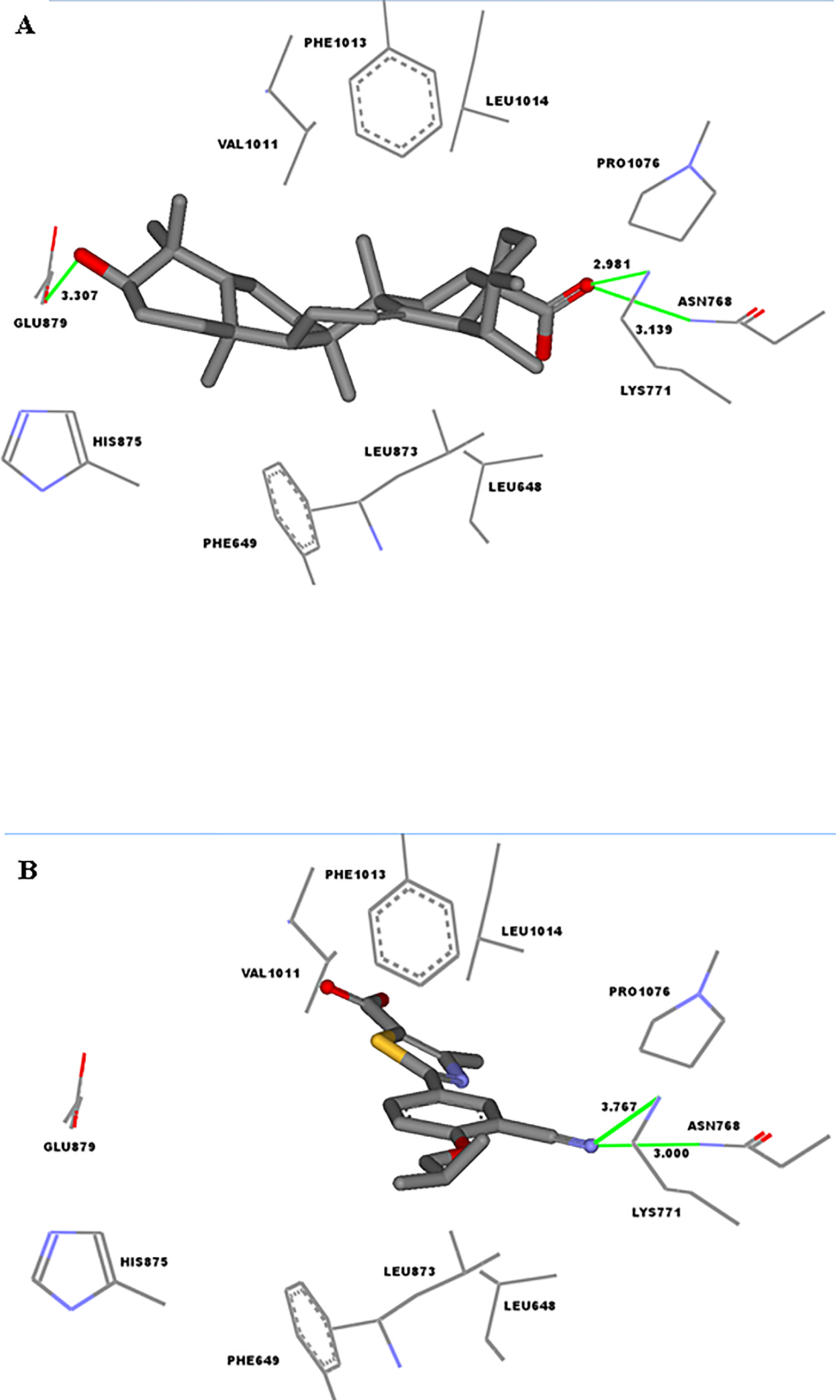
Detailed view of the binding site of bovine xanthine oxidase (XO: PDB code: 1N5X, Rs = 2.80 Ǻ) showing the key interactions with (A) docked pose of ursolic acid (IC_50_ = 10.3 ± 3.5 μg/mL), (B) co-crystallized ligand (Febuxostat, PDB code: TEI). Green lines indicate hydrogen bonding. ursolic acid and febuxostat are shown in stick representation.

In order to explore the binding mode of ursolic acid within the active site of XO, docking simulations were carried out using the docking engine FRED. Ursolic acid was successfully docked to XO with docking score value of—6.00. Interestingly, the obtained results revealed unique biding mode for ursolic acid different from the reported X-ray crystallographic determined binding modes of known XO inhibitors such as febuxostat [24], allopurinol [39] and quercetin [40] (Figs 3 and 4) in which the inhibitors bound in the channel leading from bulk solvent to the deep molybdopterin cofactor occluding it like a plug. On the other hand, ursolic acid blocks the entrance gate of channel distal from the buried molybdopterin cofactor site and extending to the bulk solvent (Fig 3C). It was emphasized that extending the interactions, as found with ursolic acid, with specific residues such as Leu-648 and Phe-649 near the gate of the channel and distal from the active site where there may be less evolutionary pressure may enhance the specificity of xanthine oxidase inhibitors [40].

A closer examination of the binding mode of ursolic acid in the active site reveals multiple favorable interactions of the natural inhibitor with active site residues near the gate of the channel. As shown in Fig 4, several hydrogen bonds were observed between ursolic acid and the XO. The most tightly bound part of ursolic acid was its carboxylate group where its oxygen atom forms a strong hydrogen bond, within 3.1 Ǻ distance, with the side chain amide of Asn-768 and charge reinforced hydrogen bonds with the terminal amino group of Lys-771 (2.9 Ǻ). The carboxylate group is located at almost the same position as the nitrile group of febuxostat, a potent XO inhibitor drug (Figs 3 and 4), which displayed a similar hydrogen bonding pattern with Asn-768 and Lys-771 (within 3.0 and 3.7 Ǻ, respectively, Fig 4B). The importance of hydrogen bonding at this particular region of the binding site was emphasized by structure–activity relationship (SAR) studies which found that the binding affinity was significantly decreased when the nitrile group of febuxostat was replaced by hydrogen [24]. On the other hand, when the cyano group of the inhibitor was replaced with a nitro group, another hydrogen bond acceptor, the binding affinity was very similar to febuxostat [41]. Moreover, the OH group of ursolic acid forms strong hydrogen bonding with Glu-879 (within 3.3 Å). Finally, the methylated hydrocarbon backbone of the pentacyclic ring system of ursolic acid lies within a hydrophobic tunnel formed from the lipophilic amino acids: Leu-648, Phe-649, Leu-873, Val-1011, Phe-1013 and Leu-1014, making substantial hydrophobic interactions within 3.0–3.3 Å distance from each side of the ursolic acid ring system and contributing to the formation of the tight ursolic acid -XO complex (Figs 3C and 4A).

Bovine XO is homologous to human enzyme with 90% overall sequence identity and the majority of the above mentioned amino acids interacting with ursolic acid are conserved among human and bovine XO [24,42]. The only exceptions are Leu648 and Phe649, which are correspond to Ile- 649 and Cys-650 in human XO, respectively. This change still preserves the lipophilic character of the side chains and their Van der Waals interaction with ursolic acid. Therefore, the binding mode suggested by molecular docking simulations would be applicable to the human enzyme.

## Conclusion

The results of this study revealed that the ethanolic crude extract of *T*. *arabicus* and its hexane fraction significantly reduced the serum uric acid levels *in vivo* and possessed high XO inhibitory activities *in vitro*. Moreover, the isolated active constituent, ursolic acid, showed potent *in vitro* XO inhibition indicating that the antihyperuricemic activity of the parent extract and its n-hexane fraction can be attributed to the isolated compound ursolic acid. *In silico* computational docking simulations also provided supportive evidence at the molecular level about the significant binding of ursolic acid with XO. Ursolic acid has good hypouricemic activity and therefore has high potential to be used for the treatment of gout and hyperuricemia-related diseases.

## Supporting information

S1 Fig ^1^H NMR spectra of ursolic acid. (PDF)

## References

[1] Landmesser U, Spiekermann S, Dikalov S, Tatge H, Wilke R, Kohler C, et al Vascular oxidative stress and endothelial dysfunction in patients with chronic heart failure: role of xanthine-oxidase and extracellular superoxide dismutase. Circulation. 2002;106: 3073–3078. 12473554 10.1161/01.cir.0000041431.57222.af

[2] Kong LD, Abliz Z, Zhou CX, Li LJ, Cheng CH, Tan RX. Glycosides and xanthine oxidase inhibitors from Conyza bonariensis. Phytochemistry. 2001;58: 645–651. 11576616 10.1016/s0031-9422(01)00176-5

[3] Unno T, Sugimoto A, Kakuda T. Xanthine oxidase inhibitors from the leaves of Lagerstroemia speciosa (L.) Pers. J Ethnopharmacol. 2004;93: 391–395. 10.1016/j.jep.2004.04.012 15234783

[4] Fried R, Fried LW. Xanthine Oxidase (Xanthine Dehydrogenase) In: Bergmeyer HU, editor. Methods of Enzymatic Analysis (Second Edition). Academic Press; 1974 pp. 644–649.

[5] Pacher P, Nivorozhkin A, Szabo C. Therapeutic effects of xanthine oxidase inhibitors: renaissance half a century after the discovery of allopurinol. Pharmacol Rev. 2006;58: 87–114. 10.1124/pr.58.1.6 16507884 PMC2233605

[6] Umpierrez A, Cuesta-Herranz J, De Las HM, Lluch-Bernal M, Figueredo E, Sastre J. Successful desensitization of a fixed drug eruption caused by allopurinol. J Allergy Clin Immunol. 1998;101: 286–287. 10.1016/S0091-6749(98)70396-3 9500766

[7] Berry CE, Hare JM. Xanthine oxidoreductase and cardiovascular disease: molecular mechanisms and pathophysiological implications. J Physiol. 2004;555: 589–606. 10.1113/jphysiol.2003.055913 14694147 PMC1664875

[8] Wallach SL. The side effects of allopurinol. Hosp Pract (1995). 1998;33: 22. 9750548

[9] Tapsell LC, Hemphill I, Cobiac L, Patch CS, Sullivan DR, Fenech M, et al Health benefits of herbs and spices: the past, the present, the future. Med J Aust. 2006;185: 4. 17022438 10.5694/j.1326-5377.2006.tb00548.x

[10] Sheng H, Sun H. Synthesis, biology and clinical significance of pentacyclic triterpenes: a multi-target approach to prevention and treatment of metabolic and vascular diseases. Nat Prod Rep. 2011;28: 543–593. 10.1039/c0np00059k 21290067

[11] Lewis CT, Short C. Latin Dictionary: Based on Andrews's edition of Freund's Latin Dictionary Oxford, New York: Oxford University Press; 1963.

[12] Chhatre S, Nesari T, Somani G, Kanchan D, Sathaye S. Phytopharmacological overview of Tribulus terrestris. Pharmacogn Rev. 2014;8: 45–51. 10.4103/0973-7847.125530 24600195 PMC3931200

[13] Akhtari E, Raisi F, Keshavarz M, Hosseini H, Sohrabvand F, Bioos S, et al Tribulus terrestris for treatment of sexual dysfunction in women: randomized double-blind placebo—controlled study. Daru. 2014;22: 40 10.1186/2008-2231-22-40 24773615 PMC4045980

[14] Al-Bayati FA, Al-Mola HF. Antibacterial and antifungal activities of different parts of Tribulus terrestris L. growing in Iraq. J Zhejiang Univ Sci B. 2008;9: 154–159. 10.1631/jzus.B0720251 18257138 PMC2225498

[15] Chhatre S, Nesari T, Somani G, Kanchan D, Sathaye S. Phytopharmacological overview of Tribulus terrestris. Pharmacogn Rev. 2014;8: 45–51. 10.4103/0973-7847.125530 24600195 PMC3931200

[16] Kamboj P, Aggarwal M, Puri S, Singla SK. Effect of aqueous extract of Tribulus terrestris on oxalate-induced oxidative stress in rats. Indian J Nephrol. 2011;21: 154–159. 10.4103/0971-4065.83727 21886973 PMC3161431

[17] Jongbloed M, Feulner G, Böer B, Western AR, Environmental Research and Wildlife, Development Agency The comprehensive guide to the wild flowers of the United Arab Emirates (in Arabic). Abu Dhabi: Environmental Research and Wildlife Development Agency; 2003.

[18] Ksiksi T, Palakkott AR, Shaijal B. Tribulus arabicus and Tribulus macropterus are Comparable to Tribulus terrestris: An Antioxidant Assessment. Current Bioactive Compounds. 2017;13: 82–87.

[19] Duong NT, Vinh PD, Thuong PT, Hoai NT, Thanh LN, Bach TT, et al Xanthine oxidase inhibitors from Archidendron clypearia (Jack.) I.C. Nielsen: Results from systematic screening of Vietnamese medicinal plants. Asian Pac J Trop Med. 2017;10: 549–556. 10.1016/j.apjtm.2017.06.002 28756918

[20] National Research Council (US) Committee for the Update of the Guide for the Care and Use of Laboratory Animals. Guide for the Care and Use of Laboratory Animals; 2011.

[21] Lorke D. A new approach to practical acute toxicity testing. Arch Toxicol. 1983;54: 275–287. 6667118 10.1007/BF01234480

[22] Lu J, Guan S, Liu J. Acute and Genetic Toxicity of Ursolic Acid Extract from Ledum pulastre L. Food Science. 2010;30: 250–252. 10.7506/spkx1002-6630-200913057

[23] Mohammad MK, Almasri IM, Tawaha K, Issa A, Al-Nadaf A, Hudaib M, et al Antioxidant, antihyperuricemic and xanthine oxidase inhibitory activities of Hyoscyamus reticulatus. Pharm Biol. 2010;48: 1376–1383. 10.3109/13880209.2010.483521 20738177

[24] Okamoto K, Eger BT, Nishino T, Kondo S, Pai EF, Nishino T. An extremely potent inhibitor of xanthine oxidoreductase. Crystal structure of the enzyme-inhibitor complex and mechanism of inhibition. J Biol Chem. 2003;278: 1848–1855. 10.1074/jbc.M208307200 12421831

[25] Discover studio (DS) visualizer. Discover studio (DS) visualizer, www.accelrys.com. 2007;2.0.

[26] MarvinSketch, calculation module developed by ChemAxon. MarvinSketch, calculation module developed by ChemAxon. 2014;6.2.2.

[27] OMEGA 2.5.1.4. OpenEye Scientific Software, Santa Fe, NM. 2013.

[28] OEDOCKING 3.2.0.2. OpenEye Scientific Software, Santa Fe, NM. 2015;3.2.0.2.

[29] Abu-Gharbieh E, Shehab NG. Therapeutic potentials of Crataegus azarolus var. eu- azarolus Maire leaves and its isolated compounds. BMC Complement Altern Med. 2017;17: 218 10.1186/s12906-017-1729-9 28420354 PMC5395866

[30] Stavric B, Clayman S, Gadd RE, Hébert D. Some in vivo effects in the rat induced by chlorprothixene and potassium oxonate. Pharmacol Res Commun. 1975;7: 117–124. 1144488 10.1016/s0031-6989(75)80015-4

[31] Jianzhen Shan Yanyan Xuan Qi Zhang Chunpeng Zhu Zhen Liu Suzhan Zhang. Ursolic acid synergistically enhances the therapeutic effects of oxaliplatin in colorectal cancer. Protein Cell. 2016;7: 571–585. 10.1007/s13238-016-0295-0 27472952 PMC4980335

[32] Pai PG, Chamari Nawarathna S, Kulkarni A, Habeeba U, Reddy C. S, Teerthanath S, et al. Nephroprotective Effect of Ursolic Acid in a Murine Model of Gentamicin-Induced Renal Damage. 2012. Available: https://www.hindawi.com/journals/isrn/2012/410902/. 10.5402/2012/410902 PMC3394390 22811930

[33] Sultana N. Clinically useful anticancer, antitumor, and antiwrinkle agent, ursolic acid and related derivatives as medicinally important natural product. J Enzyme Inhib Med Chem. 2011;26: 616–642. 10.3109/14756366.2010.546793 21417964

[34] Hu X, Shen Y, Yang S, Lei W, Luo C, Hou Y, et al Metabolite identification of ursolic acid in mouse plasma and urine after oral administration by ultra-high performance liquid chromatography/quadrupole time-of-flight mass spectrometry. RSC Adv. 2018;8: 6532–6539. 10.1039/C7RA11856B PMC9078307 35540410

[35] Zhou XJ, Hu XM, Yi YM, Wan J. Preparation and body distribution of freeze-dried powder of ursolic acid phospholipid nanoparticles. Drug Dev Ind Pharm. 2009;35: 305–310. 10.1080/03639040802302165 18798089

[36] Yang G, Yang T, Zhang W, Lu M, Ma X, Xiang G. In vitro and in vivo antitumor effects of folate-targeted ursolic acid stealth liposome. J Agric Food Chem. 2014;62: 2207–2215. 10.1021/jf405675g 24528163

[37] de Faria, Emanuelle L P, Shabudin SV, Claúdio AFM, Válega M, Domingues FMJ, Freire CSR, et al Aqueous Solutions of Surface-Active Ionic Liquids: Remarkable Alternative Solvents To Improve the Solubility of Triterpenic Acids and Their Extraction from Biomass. ACS Sustainable Chem Eng. 2017;5: 7344–7351. 10.1021/acssuschemeng.7b01616 PMC6157723 30271685

[38] [Anonymous]. Application of ursolic acid to preparation of medicine for treating gout.; CN 201611247667.

[39] Okamoto K, Eger BT, Nishino T, Pai EF, Nishino T. Mechanism of inhibition of xanthine oxidoreductase by allopurinol: crystal structure of reduced bovine milk xanthine oxidoreductase bound with oxipurinol. Nucleosides Nucleotides Nucleic Acids. 2008;27: 888–893. 10.1080/15257770802146577 18600558

[40] Cao H, Pauff JM, Hille R. X-ray crystal structure of a xanthine oxidase complex with the flavonoid inhibitor quercetin. Journal of natural products. 2014;77: 1693 10.1021/np500320g 25060641

[41] Hille R, Massey V. Molybdenum Enzymes. New York: John Wiley and Sons; 1985.

[42] Berglund L, Rasmussen JT, Andersen MD, Rasmussen MS, Petersen TE. Purification of the Bovine Xanthine Oxidoreductase from Milk Fat Globule Membranes and Cloning of Complementary Deoxyribonucleic Acid1. Journal of Dairy Science. 1996;79: 198–204. 10.3168/jds.S0022-0302(96)76351-8 8708081

