# Supplementary figures and images for: Antihyperuricemic and xanthine oxidase inhibitory activities of Tribulus arabicus and its isolated compound, ursolic acid: In vitro and in vivo investigation and docking simulations
Source: PLoS One. 2018 Aug 16;13(8):e0202572. doi: 10.1371/journal.pone.0202572 (PMC6095567; doi:10.1371/journal.pone.0202572)

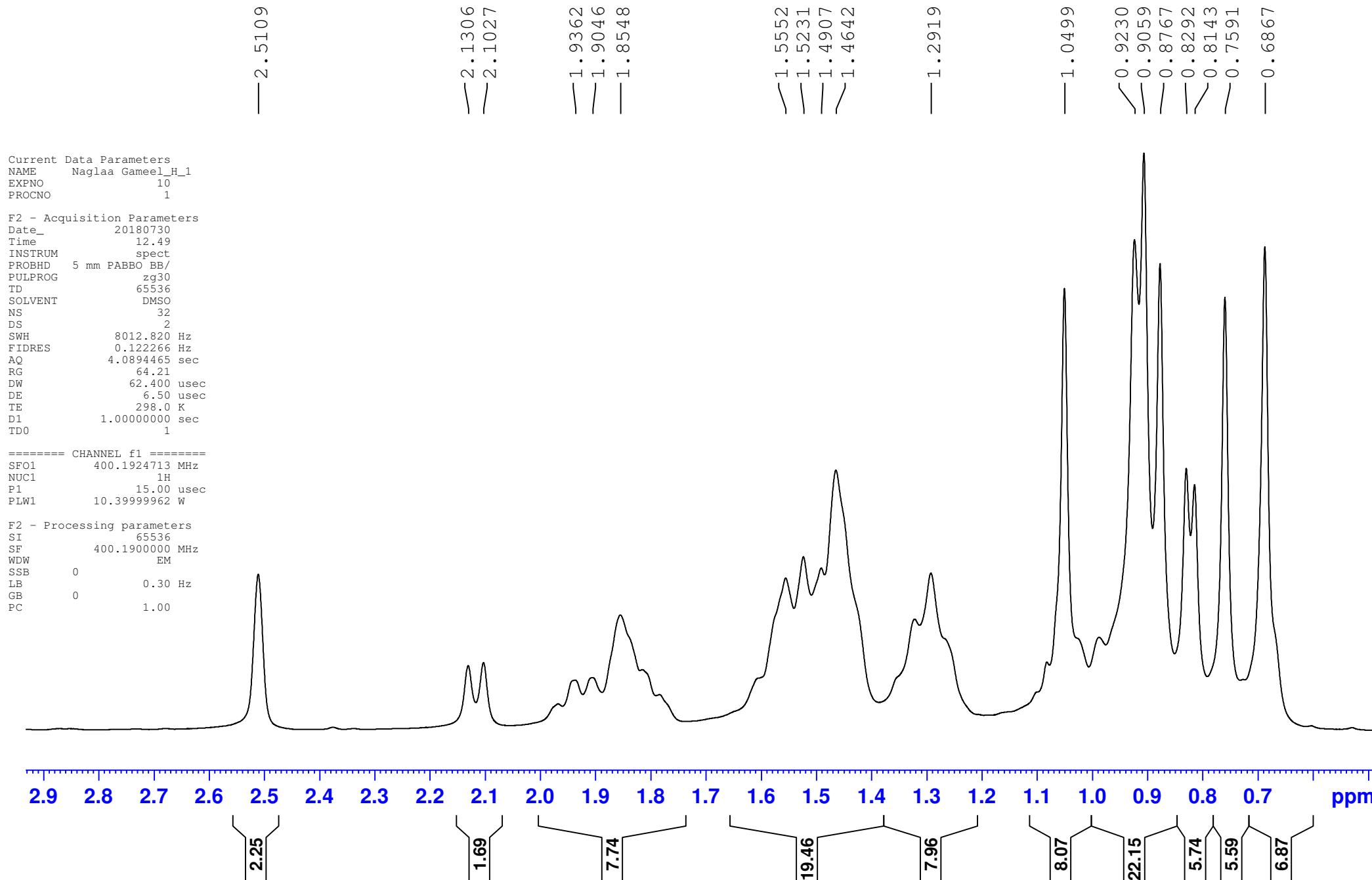S1 Fig. <sup>1</sup>H NMR spectra of ursolic acid using DMSO as solvent

Supplement: S1 Fig — (PDF) [file pone.0202572.s001.pdf]
